# The growth inhibitory effects of cadmium and copper on the MDA-MB468 human breast cancer cells

**Published:** 2010

**Authors:** Mojtaba Panjehpour, Masih-Allah Taher, Mortaza Bayesteh

**Affiliations:** a Department of Clinical Biochemistry, School of Pharmacy, Isfahan Pharmaceutical Sciences Research Center, Isfahan University of Medical Sciences, Isfahan, Iran

**Keywords:** Elements, Trace, Toxic Potential, Breast Neoplasm, Cadmium, Copper

## Abstract

**BACKGROUND:**

Cadmium chloride is an important occupational and environmental pollutant. However, it can also be anti-carcinogenic under certain conditions. Copper, an essential trace element, has the ability to generate reactive oxygen species and induce cell apoptosis. This study was aimed to determine the growth inhibitory effects of cadmium and copper on the MDA-MB468 human breast cancer cells.

**METHODS:**

By using MTT cell viability test, treatment of monolayer cell cultures with different metal concentrations (1-1000 µM) showed a significant dose dependent decrease (p < 0.05) of viable cells in different times.

**RESULTS:**

A considerable cytotoxicity was observed for CdCl2 at 200 µM and 1 µM after 48 and 72 hours incubations, respectively. The highest concentration of CuCl2 (1000 µM) had little cytotoxic effects after 48 hours incubation period, but 1 µM of CuCl2 revealed a considerable cytotoxicity after 72 hours. The maximum synergic cytotoxic effect was observed at 0.5 µM of both metals.

**CONCLUSIONS:**

The results of the present study indicate that cytotoxic effect of CuCl2 is somehow lesser than that of CdCl2. This may be due to vital role of copper which is not known for cadmium so far.

Cadmium, a heavy metal, has been known to be harmful to human health, due to its wide use and its increasingly accumulation in the environment, mainly via contaminated drinking water, food supplies, tobacco, and agricultural and industrial pollutants. In the human body, cadmium has a biological half-life of more than 20 years. Therefore it is not surprising that it causes damage in several organs including the lung, liver, kidney, cardiovascular, hematopoietic, immune and nervous systems due to occupational intoxication.^1^–^5^ It has also been demonstrated to be a cytotoxic agent that induces cell death either by necrotic or apoptotic mechanisms. Cadmium toxicity has been shown to affect cells through very complex functions such as Reactive Oxygen Species (ROS) production and oxidative damage, interfering with homeostasis of calcium, copper or zinc,^6^ lysosomal damage, lipid peroxidation, disruption of mitochondrial function,^7^,^8^ direct interaction with DNA, alterations in proto-oncogene expression, inhibition of DNA repair processes, and induction of apoptosis.^9^

In spite of these findings, other studies indicate that cadmium can suppress apoptosis in vivo. Several evidences demonstrate the carcinogenicity of cadmium in experimental animal models as well as the clear association between occupational cadmium exposure and human cancers including pulmonary, renal, prostate, hematopoietic system, liver, stomach, urinary bladder and pancreas cancers. Therefore cadmium has been classified as a ubiquitous environmental carcinogen by International Agency for Research in Cancer (IARC) and the US National Toxicology Program.^10^–^12^ Copper, an essential trace element, is a cofactor of various proteins and enzymes involved in oxygen metabolism. Although excess copper ions are known to lead to necrosis or apoptosis through ROS production and oxidation of lipids or proteins^13^ via Fenton or Haber-Weiss reactions,^14^ the antioxidant properties of copper ions involve it in the defense against ROS.^15^ It binds to DNA and induces formation of strand breaks and chromosomal aberrations. High concentrations of copper also have carcinogenic effects^4^ and induce growth proliferation and cancer by damaging DNA with ROS.^16^ Chronic copper exposure causes serious liver disorders and can damage the gastrointestinal tract, brain, renal tubules, and coronary artery.^4^

Despite improvements in both breast cancer prevention and treatment, the mechanism by which the trace elements may be involved in the initiation of the breast cancer is not known.^17^–^19^

The main aim of this study is to determine the relative cytotoxic effects of cadmium chloride and copper chloride, alone and in combination, in cultures of the human breast cancer cell line, MDA-MB468.

## Methods

### 

#### Chemicals

All culture media, reagents and growth supplements were purchased from Gibco (Scotland). Flasks, petri dishes and multi-well plates were obtained from Nunc (Denmark). CdCl2 and CuCl2 were from Merck (Germany). All other chemicals were of analytical grade and were purchased from Sigma Aldrich (St. Louis, MO, USA) or were of the highest purity commercially available.

#### Cell Lines and Cell Culture Conditions

The human breast cancer cell line, MDA-MB468, was obtained from Pasture Institute of Iran. The cell line was grown in RPMI-1640 media supplemented with heat-inactivated 10% fetal bovine serum, 100 U/ml penicillin, and 100 µg/ml streptomycin. The cells were maintained in a humidified atmosphere at 37°C in 5% CO_2_/95% air. Cells were passaged two or three times weekly and examined regularly for contamination and growth assessment. The cells were cultured in 25 cm^2^ cell culture flasks. For experimental purposes, cells were cultured in 96 multi-well plates. The optimum cell concentration as determined with the growth profile of the cell line was 30,000 cells/ml.

#### Drug Treatment

The cells (30,000 cells/ml) were plated and allowed to attach. After 24 hours, the medium was replaced with fresh medium. CdCl_2_ and CuCl_2_ were dissolved in water and the stock solutions were sterilized by filtration and then further diluted with medium to the desired concentrations. Different concentrations of cadmium chloride and copper chloride (1-1000 µM) were added to the plates which were incubated for 48 and 72 hours. The concentration range and the exposure times have been selected based on our preliminary works.

#### Cell Viability Assay

The effects of different concentration of CdCl_2_ and CuCl_2_ on the number of living cells were determined using the MTT-assay. MDA-MB468 human breast cancer cells were cultured in 96 well plates with or without the added test metal ions. At the end of incubation MTT (3-[4, 5-dimethylthiazol-2-yl]-2, 5-diphenyltetrazolium bromide) solution was added to each well (0.5 mg/ml) and incubation continued further for 4 additional hours to determine the extent of cytotoxicity caused by test chemicals. Cells having functional mitochondrial succinate dehydrogenase can convert MTT to formazan. Then the medium was removed and DMSO was added to dissolve the formazan crystals to blue color. The optical density of samples was read on an ELISA microplate reader at 570 nM. The color formation and absorbance were directly proportion to the number of living cells. The results of cell viability are expressed as percent of untreated control cells. Cell viability is shown graphically as percent of the control cells.

#### Statistical Analysis

Non parametric one-way analysis of variance (ANOVA) was performed followed by post-hoc Dunnett’s test, using SPSS software, v. 11.0. Each experiment was carried out in triplicate and repeated three to four times independently. Differences were considered statistically significant when a p < 0.05 was achieved. All data in the figures and text are presented as means ± standard deviation (SD) of n observations (with n > 9).

## Results

### 

#### The Cytotoxic Effects of Cadmium on Human Breast MDA-MB468 Cell Line

**Figure 1:**
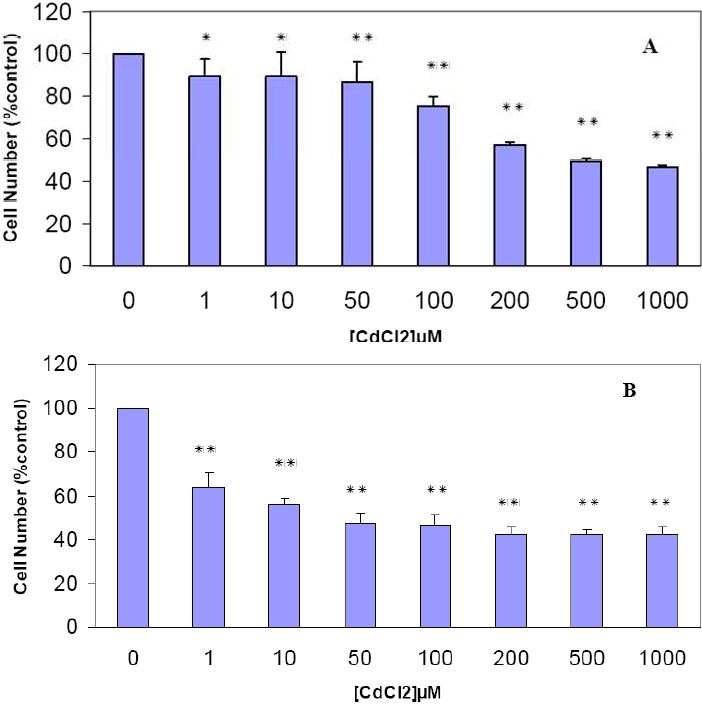
The cytotoxic effect of CdCl_2_ on human breast MDA-MB468 cell line. The cells were incubated with CdCl_2_(1-1000 µM). After 48 hours (A) and 72 hours (B), an MTT cytotoxicity assay was performed as described in “Methods.” Results are mean ± SD of three separate experiments. * P < 0.05 (significant difference from control) ** P < 0.01 (significant difference from control)

**Figure 2:**
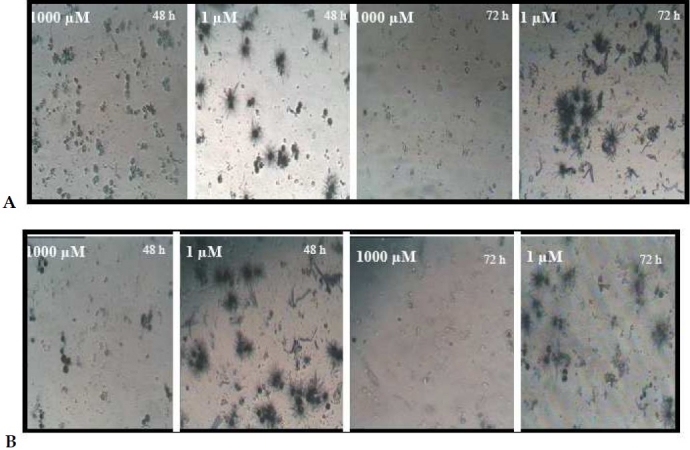
The morphological changes of MDA-MB-468 cells after MTT assay The cells were treated with low and high concentrations of CdCl_2_(A) and CuCl_2_(B) and followed by MTT assay. Morphological study by inverted microscope revealed insoluble formazan crystal formation that proportions directly to the number of living cells.

MTT cell viability test was used to study the cytotoxic effects of cadmium chloride. Treatment of cultured monolayer cells with different metal concentrations (1-1000 µM) showed a significant time-dependent decrease (p < 0.05) of viable cells when compared with that of controls. Therefore a dose and time dependent toxicity was found for CdCl_2_ (Figure 1). For example, a mild cytotoxicity was observed at 1 µM of CdCl_2_, and cell growth was reduced to 89% (Figure 1A) of the control after 48 hours incubation and reached to the maximum at higher concentrations. Cadmium chloride concentration of 200 µM at 48 hours was found to be more toxic for the cells and reduced cell viability to about 60% (Figure 1A). Increasing of the incubation time resulted in increased toxicity. As shown in figure 1B, peak of cytotoxicity of CdCl_2_ was shifted to the lower micromolar concentration at 72 hours and cell viability was reduced to 63% at 1 µM concentration of CdCl2. However greater concentrations had no significant effect to accelerate the power of cytotoxicity. The morphological appearance of the cells exposed to MTT after treatment with low and high concentrations of CdCl_2_ is shown in figure 2A.

#### The Viability of Human Breast MDA-MB468 Cells Treated With CuCl_2_

**Figure 3:**
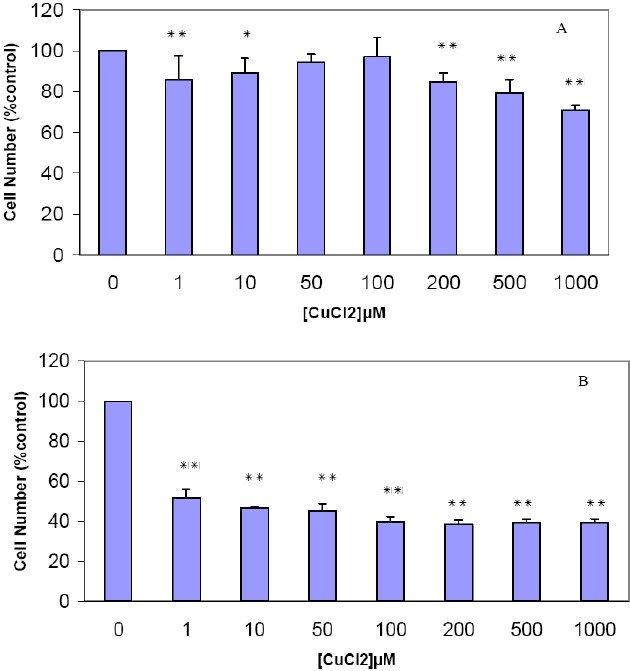
The effects of CuCl_2_ on the viability of human breast MDA-MB468 cell line The Cells were treated with CuCl_2_ (1-1000 µM). After 48 hours (A) and 72 hours (B), an MTT cytotoxicity assay was performed as described in “Methods.” Results are mean ± SD of three separate experiments. * P < 0.05 (significant difference from control) ** P < 0.01 (significant difference from control)

To evaluate the cytotoxicity of CuCl _2_, the effects of CuCl_2_ on the growth rate and viability of cultured human breast MDA-MB468 cells was measured. The cells were incubated with increasing doses of CuCl_2_ for 48 and 72 hours, and effects on viability were assessed using an MTT dye reduction assay. As seen in figure 3A, after 48 hours, about 70% of the cells survived at 1000 µM Cu exposure. Thus, a high concentration of CuCl2 was tolerable and the cell growth was slightly inhibited by this metal ion. Its toxicity was markedly increased by increase of incubation time from 48 hours to 72 hours. Figure 3B shows that the growth of 1 µM Cu treated breast cancer cells was markedly inhibited after 72 hours and approximately 52% of cells survived after exposure to 1 µM of Cu. Maximum growth inhibition was reached at higher concentration after 72 hours incubation. The morphological appearance of copper chloride induced cytotoxicity was similar to that of cadmium (Figure 2B).

#### The Combined Cytotoxic Effects of CdCl_2_ and CuCl_2_

**Figure 4:**
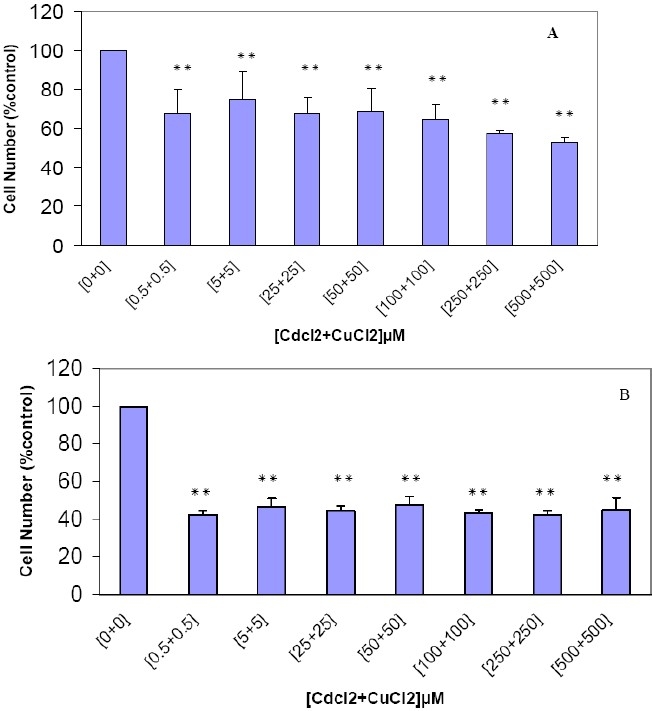
The combined cytotoxic effects of CdCl_2_ and CuCl_2_ on human breast MDA-MB468 cell line. The cells were incubated with varying concentrations of CdCl_2_ and CuCl_2_ for 48 hours (A) and 72 hours (B) and viability was assessed using MTT assay as described in the “Methods.” Results are mean ± SD of three separate experiments. * P < 0.05 (significant difference from control) ** P < 0.01 (significant difference from control)

The effect of simultaneous addition of cadmium chloride and copper chloride on MDA-MB468 cell viability was also assessed using the MTT assay. As shown in figure 4, the cytotoxic effect of the simultaneous administration of the same concentrations of the two metal ions is more potent than addition of each metal separately. In a time and dose-dependent manner, the maximum synergic cytotoxic effects was observed at 0.5 µM cadmium chloride and 0.5 µM copper chloride during 72 hours exposure (Figure 4B).

## Discussion

The present study was conducted to study the effects of cadmium and copper chloride, alone and in combination, on human breast cancer cell line, MDA-MB468. The results clearly indicate that cadmium and copper chloride are highly cytotoxic to the cells in a dose and time dependent manner. A considerable cytotoxicity was observed at 200 µM for cadmium chloride during 48 hours and 1 µM during 72 hours. While high concentration of CuCl2 was tolerable in 48 hours treatment, its toxicity was markedly increased and shifted to lower concentration (1 µM) during 72 hours incubation. The maximum synergic cytotoxic effect was observed at 0.5 µM cadmium chloride and 0.5 µM copper chloride during 72 hours exposure.

The presence of urinary cadmium in 5% of the US population demonstrates the need for more researches that address the role of cadmium in human health.^9^ Interestingly the human mammary gland also contains high concentrations of cadmium that mimics the effects of estradiol and might play a putative role in the etiology of the breast cancer.^17^–^19^

The results from the present study are in agreement with previous studies. Cadmium induces apoptosis in various tissues and cells, e.g. mouse liver and thymocytes, rat testis, lung epithelial and fibroblast cells, human lung and cervix carcinoma cells, human kidney, myocardial, glioma, neuroblastoma, prostate, smooth muscle and T cells.^19^–^21^ Cadmium-induced apoptosis is inhibited by metal-lothionein, which binds to cadmium to prevent toxic damage.^1^

The molecular mechanism of cadmium induced apoptosis/necrosis or its relationship to ROS production remains controversial. Although antioxidative agents did not protect against cadmium-induced apoptosis in rat alveolar cells, cadmium-induced apoptosis was inhibited by antioxidant in U-937 cells. Moreover, glutathione or catalase prevented cadmium-induced apoptosis in rat C6 glioma cells.^11^,^20^ Cadmium induces apoptosis in HL60 leukemia cells and C6 rat glioma cells through activation of caspase^9^ or independent of caspase activity. Moreover, it was shown that cadmium inactivated the p53 protein in human breast cancer MCF-7 cells.^11^ It has been suggested that cadmium disrupts mitochondrial function^22^ and induces lysosomal damage^8^ as first targets which leads to other cellular events including DNA damage. It has been shown that cadmium causes cell death by induction of apoptosis. To confirm the oxidative hypothesis of cadmium cytotoxicity, pre treatment of the MDA-MB468 cells with N-acetylcysteine, a free radical scavenger, was carried out. N-acetylcysteine administration reversed the cadmium cytotoxic effects and protected cells from apoptotic death. So, induction of apoptosis caused by CdCl2 is mediated, by ROS production.

In experimental animal models, cadmium compounds have been shown to induce various tumors.^21^ It is thought that cadmium acts via genotoxic mechanisms such as induction of stranded DNA breaks, increasing mutation rate, and activation of proto-oncogenes as well as inhibition of DNA repair and apoptosis.^8^,^11^,^12^,^17^,^19^,^21^ The cadmium carcinogenicity seems to be mediated through the production of ROS.^11^ However, the different conditions and sensitivities of the cells are the main explanations for both inhibitory and stimulatory effects of cadmium. It was suggested that the antiapoptotic effects of cadmium is due to induction of metalothionin expression in the cell, but free cadmium ion triggers the induction of apoptosis.^4^

Pro-oxidant and antioxidant properties of copper ions involve it in both the generation of and the defense against ROS in biological systems.^15^ This paradoxical effect of copper makes it to become toxic at high concentration. This cytotoxicity of copper has been applied to cancer chemotherapy. It has been shown that Cu^2+^ induces apoptosis in a dose and time dependent manner in mouse pro-B cell line BA/F3β via upregulation of Bax and ROS and subsequent inactivation of NF□B.^9^ Damage to microsomal proteins can be explained solely by the Cu^2+^-binding effect (not by ROS generation).^14^ Schmidt et al showed a protective effect of Cu at low concentrations in MCF-12A cells (a non tumorogenic breast cell line) that is not observed in tumorogenic MDA-MB231 cells.^23^ Rossi et al suggested that the cytotoxic effects of cadmium in Caco-2 human colon carcinoma cell line are more than copper.^24^ This observation is similar to the present findings in this study. It has been reported that cadmium-zinc combinations at low concentration have a synergistic cytotoxic effect in vitro.^13^,^25^ Furthermore it has been shown that the cytotoxic effects of CuCl2 and CdCl2 on the hepatocyte cells occur as a result of a mitochondrial ROS formation due to their ability to decrease mitochondrial membrane potential rather than their ability to catalyze hepatocyte ROS formation, lipid peroxidation or GSH oxidation.^7^

## Conclusions

According to the findings of this study, it is concluded that there is a different sensitivity of the cell line to antitumor activity of cadmium chloride and copper chloride. The results of the present study also indicate that cytotoxic effect of copper chloride is somehow less than that of cadmium chloride. This may be due to vital role of copper which is not known for cadmium so far. In other words, copper can also be used for natural consumption of the cells. A comprehensive study to clarify the molecular mechanism of cadmium induced apoptosis/necrosis or its relationship to ROS production in different human breast cancer cell lines is in progress.
